# Establishment of HIV-negative neurosyphilis risk score model based on logistic regression

**DOI:** 10.1186/s40001-023-01177-5

**Published:** 2023-06-29

**Authors:** Yu Fu, Ling Yang, Jie Du, Raqib Khan, Donghua Liu

**Affiliations:** 1 Department of Dermatology and Venereology, First Affiliated Hospital of Guangxi Medical University, No.6 Shuangyong Road, Nanning, 530021 China; 2 Department of Biostatistics, School of Public Health, Guangxi Medical University, Nanning, China

**Keywords:** Treponema pallidum, Neurosyphilis, Logistic regression analysis, Risk factors, Risk score model

## Abstract

**Objective:**

To establish the risk scoring model for HIV-negative neurosyphilis (NS) patients and to optimize the lumbar puncture strategy.

**Methods:**

From 2016 to 2021, clinical information on 319 syphilis patients was gathered. Multivariate logistic regression was used to examine the independent risk factors in NS patients who tested negative for human immunodeficiency virus (HIV). Receiver operating characteristic curves (ROC) were used to assess the risk scoring model’s capacity for identification. According to scoring model, the timing of lumbar puncture was suggested.

**Results:**

There were statistically significant differences between HIV-negative NS and non-neurosyphilis (NNS) patients in the following factors. These included age, gender, neuropsychiatric symptoms (including visual abnormalities, hearing abnormalities, memory abnormalities, mental abnormalities, paresthesia, seizures, headache, dizziness), serum toluidine red unheated serum test (TRUST), cerebrospinal fluid Treponema pallidum particle agglutination test (CSF-TPPA), cerebrospinal fluid white blood cell count (CSF-WBC), and cerebrospinal fluid protein quantification (CSF-Pro) (*P* < 0.05). Logistic regression analysis of HIV-negative NS patients risk factors showed that age, gender, and serum TRUST were independent risk factors for HIV-negative NS (*P* = 0.000). The total risk score (− 1 ~ 11 points) was obtained by adding the weight scores of each risk factor. And the predicted probability of NS in HIV-negative syphilis patients (1.6 ~ 86.6%) was calculated under the corresponding rating. ROC calculation results showed that the score had good discrimination value for HIV-negative NS and NNS: area under the curve (AUC) was 0.80, the standard error was 0.026 and 95% CI was 74.9–85.1% (*P* = 0.000).

**Conclusion:**

The risk scoring model in this study can classify the risk of neurosyphilis in syphilis patients, optimize the lumbar puncture strategy to a certain extent, and provide ideas for the clinical diagnosis and treatment of HIV-negative neurosyphilis.

## Introduction

Syphilis is a chronic infectious disease with a long history. In the middle of the 1490s, it was first mentioned in Europe. It results from *T. pallidum* subspecies infection. Nowadays, the incidence of syphilis is gradually increasing. According to the statistics of the World Health Organization (WHO), the prevalence of syphilis in developing countries is greater than that in developed countries [1]. Syphilis has become an international concern because of its high infectivity and relatively poor prognosis. New developments in syphilis are also emerging in the study of infectious diseases. Studies have shown that the human immunodeficiency virus (HIV)-positive population is more likely to be infected with syphilis than the general population [2]. At the same time, the spread of syphilis also led to the development of various sexually transmitted diseases, including HIV infection. Therefore, many patients infected with syphilis were also infected with other sexually transmitted pathogens. This trend consolidated the position of syphilis in the field of sexually transmitted diseases.

Neurosyphilis (NS) is a series of neuropsychiatric syndromes caused by *T. pallidum* invasion of the brain parenchyma or meninges [3]. NS is generally considered to be a late syphilis disease. However, recent studies have found that NS can occur at any stage of the course of syphilis [4, 5]. So far, there is no gold standard for the diagnosis of NS, which makes the timely diagnosis and treatment of NS patients a great challenge. Lumbar puncture and cerebrospinal fluid (CSF) examination have become important methods to assist in the diagnosis of NS. However, CSF examination is an invasive examination method, and there is no set rule for whether to perform lumbar puncture or which patients should have lumbar puncture. Thus, it is critical to think about how to evaluate NS risk in syphilis patients, timely targeted screening of high-risk individuals, and reducing the rate of missed diagnoses. The purpose of this study was to establish a method to digitize and stratify the risk of neurosyphilis in syphilis patients on the basis of a retrospective study so as to optimize the lumbar puncture strategy. Thus, by reducing missed diagnoses and misdiagnoses, it can provide ideas for clinical diagnosis and improve medical efficiency.

## Methods

### Objects

1. A total of 319 inpatients with syphilis were admitted from 2016 to 2021. The clinical information and laboratory results of the patients were collected from the hospital system.
2. Clinical diagnostic criteria for syphilis patients: A positive serum Treponema pallidum particle agglutination (TPPA) is clinically diagnosed in syphilis patients, including all patients with a positive or negative toluidine red unheated serum test (TRUST).

### Laboratory methods

Laboratory detection of syphilis, including the TPPA test (Fujirebio, Tokyo, Japan) and serum TRUST (Rongsheng Biotech, Shanghai, China), was conducted as per the manufacturers’ instructions. All the above methods were used for CSF and serum analysis.

### Inclusion criteria

(1) The results of serum TPPA, serum TRUST, CSF-TPPA quantification, CSF-TRUST qualitative and quantitative, CSF-white blood cell count (CSF-WBC), and CSF protein quantification (CSF-Pro) were complete. (2) HIV-negative patients.

### Diagnostic criteria for HIV-negative NS patients [5–9]

Patients with neurosyphilis are divided into symptomatic and asymptomatic neurosyphilis according to whether they are accompanied by signs and symptoms of neurosyphilis (which include visual abnormalities, hearing abnormalities, memory loss, mental and behavioral abnormalities, paresthesia, seizures, headaches, dizziness, tabes, paralytic dementia, cerebrovascular neurosyphilis, and gummy neurosyphilis).

1. Diagnostic criteria for symptomatic neurosyphilis: ① concomitant signs and symptoms of neurosyphilis; ② CSF-TPPA (+); ③ CSF-Pro > 450 mg/L and CSF-WBC > 5/μL. The first of these is the required diagnostic condition, while the remaining two conditions can be present simultaneously or separately.
2. Diagnostic criteria for asymptomatic neurosyphilis: ① no signs and symptoms of neurosyphilis; ② CSF-TPPA (+) and CSF-Pro > 450 mg/L and CSF-WBC > 5/μL. The above two conditions are mandatory diagnostic conditions and must occur simultaneously.

Neurosyphilis patients with the above diagnosis combined with HIV-negative infection were defined as HIV-negative NS patients.

### The clinical data of 319 HIV-negative syphilis patients were retrospectively analyzed

Data were collected in the WPS Office, and SPSS 25.0 was used to analyze the data. According to the diagnostic criteria for HIV-negative NS, the patients were divided into the NS group and the NNS group. The concentration and dispersion of normal-distribution continuous variables were expressed as mean ± standard deviation and analyzed by *T*-test. The degree of concentration and dispersion of skewed continuous variables were expressed by median and interquartile distance and analyzed by the rank sum test. Categorical variables were expressed as frequency and component ratio and analyzed by a Chi-square test. *P* > 0.05 was statistically significant.

### Methods of risk classification

With reference to previous literature, the risk of disease was divided into three levels from low to high: low-risk (incidence ≤ 10.0%), medium-risk (incidence ≤ 20.0%) and high-risk (incidence > 20.0%) [10]. According to the incidence rate and risk grading of diseases in the cited literature, combined with the realistic incidence rate and risk score of neurosyphilis in this study, the risk grading of neurosyphilis in this study was calculated.

## Results

Clinical data and analysis results of 319 syphilis patients showed that HIV-negative NS and NNS in age, gender, neuropsychiatric signs and symptoms (including visual abnormalities, hearing abnormalities, memory loss, mental and behavioral abnormalities, paresthesia, seizures, headaches, dizziness), serum TRUST qualitative and quantitative, CSF-TPPA qualitative, CSF-WBC, and CSF-Pro were statistically different (*P* > 0.05) (Table 1).

**Table 1:** General clinical information of 319 patients with syphilis

Variate	NS (93)	NNS (226)	*t/χ*^2^*/Z*	*P*
Age (year)	49.24 ± 10.99	39.70 ± 15.07	66.289	0.000
Gender			31.593	0.000
Male	74 (76.6)	102 (45.1)		
Female	19 (20.4)	124 (54.9)		
Neuropsychiatric symptoms and signs			141.130	0.000
Yes	87 (93.5)	48 (21.2)		
No	6 (6.5)	178 (78.8)		
Serum TRUST quantitate			22.286	0.000
Negative	11 (11.8)	60 (26.5)		
1:1 ~ 1:2	26 (28.0)	87 (38.5)		
1:4 ~ 1:8	25 (26.9)	45 (19.9)		
1:16 ~ 1:32	24 (25.8)	22 (9.7)		
≥ 1:64	7 (7.5)	12 (5.3)		
Serum TRUST qualitative			8.251	0.004
Positive	82 (88.2)	166 (73.5)		
Negative	11 (11.8)	60 (26.8)		
CSF-TPPA quantitate			133.452	0.000
Positive	88 (94.6)	54 (23.9)		
Negative	5 (5.4)	172 (94.6)		
CSF-WBC (/μL)	6.00 (29.00)	0.00 (2.00)	− 6.504	0.000
CSF-Pro (mg/L)	473.50 (249.35)	292.80 (136.85)	− 8.837	0.000

Logistic regression analysis of risk factors for HIV-negative NS showed that age, gender, and serum TRUST qualitative were independent risk factors for HIV-negative NS (*P* = 0.000) (Table 2).

**Table 2:** Logistic regression analysis of HIV-negative NS patients

Variate	*β*	*SE (β)*	*Wald*	*P*	*OR* (*95% CI*)
Age (year)^a^	0.050	0.010	24.161	0.000	1.051 (1.031 ~ 1.073)
Serum TRUST quantitate^b^	0.502	0.121	17.151	0.000	1.651 (1.302 ~ 2.094)
Gender (male)	1.431	0.311	21.199	0.000	4.184 (2.275 ~ 7.695)
Constant (*C*)	− 4.823	0.614	61.647	0.000	0.008

^a^The age group

^b^Serum TRUST qualitative group. Reference Table 1

The weight score of each independent risk factor (*Points*_*ij*_) was calculated: the age from low to high was 1–4 points, the female was 0 points, the male was 3 points, and the serum TRUST quantification from low to high was 0 to 4 points (Table 3).

**Table 3:** HIV-negative NS risk factor weight rating scale

Risk factors	Groups	*W*_*ij*_	*β*_*i*_	*D*	*Points*_*ij*_ (Points_*ij*_)
Age (year)			0.050		
	< 20	15.5		− 0.450	− 1
	20 ~ 29	24.5 = *W*_*1REF*_		0.000	0
	30 ~ 39	34.5		0.500	1
	40 ~ 49	44.5		1.000	2
	50 ~ 59	54.5		1.500	3
	≥ 60	68.5		2.200	4
Gender			1.431		
	Female	0 = *W*_*2REF*_		0	0
	Male	1		1.431	3
Serum TRUST quantitate^a^			0.502		
	0	0 = *W*_*3REF*_		0.000	0
	1	1		0.502	1
	2	2		1.004	2
	3	3		1.506	3
	4	4		2.008	4

^a^0 indicates that the serum TRUST is qualitatively negative; 1 indicates that the serum TRUST quantitate is 1:1 ~ 1:2; 2 indicates 1:4 ~ 1:8; 3 indicates 1:16 ~ 1:32, and 4 indicates ≥ 1:64

*D* = (*W*_*ij*_ − *W*_*iREF*_) * *β*_*i*_

*B* = *5* * *β*_*i*_

*Points*_*ij*_ = *D/B*

**Fig. 1:**
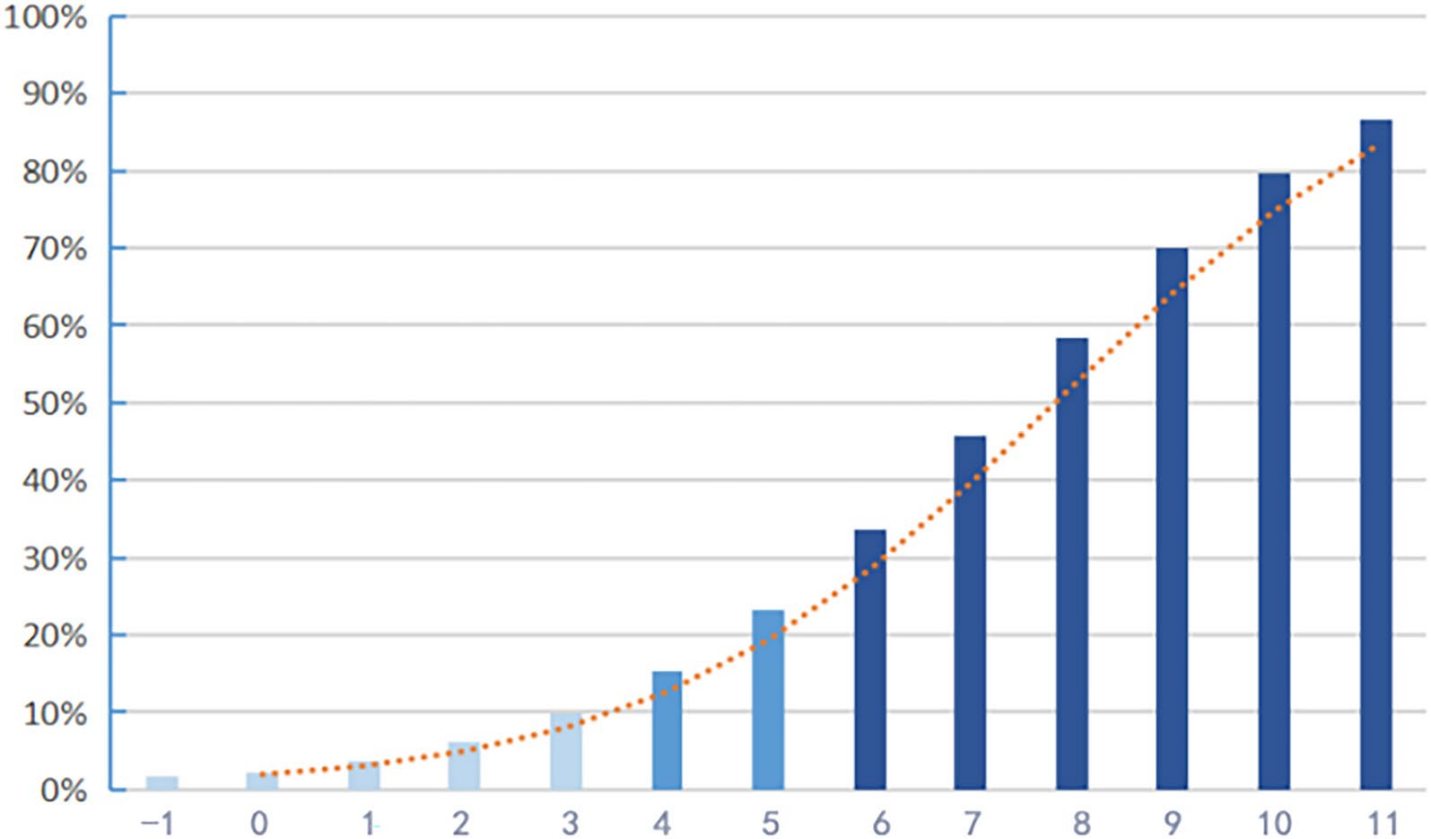
HIV-negative NS risk probability trend chart. The ordinate represents the risk prediction probability; the abscissa represents the risk score

The total risk score (− 1 ~ 11 points) was obtained by adding the weight scores of each risk factor, and the predicted probability of NS in HIV-negative syphilis patients was calculated (1.6–86.6%) under the relative score. The prevalence and risk classification were compared, and the calculation was as follows: low-risk = 29.2% * 10%/20.4% ≈ 14% (− 1 ~ 3 points), medium-risk = 29.2% * 20%/20.4% ≈ 27% (4 ~ 5 points), and high-risk ≥ 27% (6 ~ 11 points) (Table 4 and Fig. 1).

**Table 4:** HIV-negative NS risk score and predicted probability

Risk score (*S*)	Predicted probability ($\hat{P}$)	Risk classification
− 1	0.016334604	Low risk
0	0.023146344	
1	0.037960842	
2	0.061658665	
3	0.098633588	
4	0.154139033	Medium risk
5	0.232812111	
6	0.335703557	High risk
7	0.456981619	
8	0.583583116	
9	0.700042448	
10	0.795353082	
11	0.866168742	

$$\hat{P} = 1/\left[ {1 + \exp \left( { - \mathop \sum \limits_{i = 0}^{P} \beta_{i} X_{i} } \right)} \right]$$

**Fig. 2:**
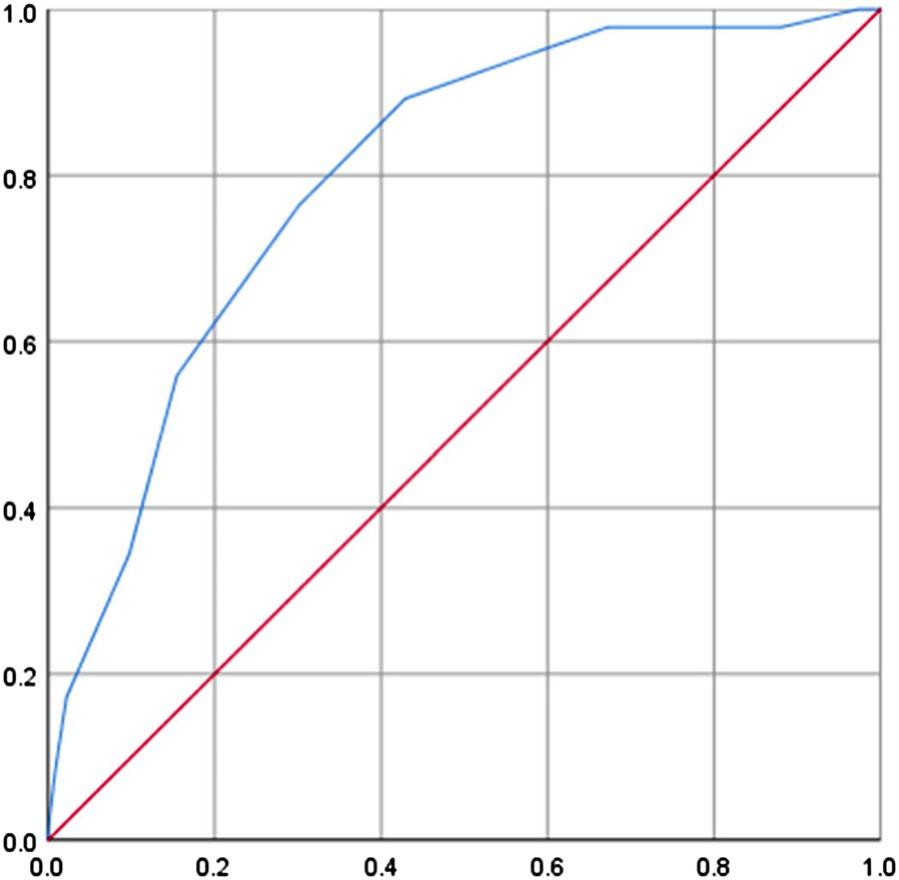
ROC curve of HIV-negative NS risk score. The ordinate is sensitivity, and the abscissa is (1 − specificity)

Receiver operating curve (ROC) calculation results showed that the model had a good discrimination value for HIV-negative NS and NNS: AUC = 0.80, standard error = 0.026, 95% CI was 74.9–85.1%, *P* = 0.000 (Fig. 2).

## Discussion

There has been a lack of a gold standard for the diagnosis of NS for a long time. Lumbar puncture and laboratory examination of the CSF have become important means to assist in the diagnosis of NS. For symptomatic NS, clinical manifestations and CSF examination results can be combined to make a judgment. Many studies have examined the use of CSF in the Venereal Disease Research Laboratory (VDRL) to diagnose NS [5, 6]. In 2012, Weiming GU et al. believed that although CSF-TRUST could be selected to diagnose NS without CSF-VDRL, the sensitivity of CSF-TRUST was still poor compared with that of CSF-VDRL [7]. However, in a 2021 study by Yong Lu et al., it was directly shown that CSF-TPPA can be considered an alternative test for the diagnosis of neurosyphilis, with ROC analysis showing a sensitivity of 90%, a specificity of 84%, and an AUC of 0.941 for CSF-TPPA titers. CSF-TPPA titers, when combined with neuropsychiatric symptoms, CSF-protein, and CSF-WBC, have a high sensitivity for diagnosis [8]. In the 2020 European guideline on the management of syphilis, it was mentioned that although a positive CSF-TPPA cannot confirm the diagnosis of neurosyphilis, the specificity of the detection is high, so when the CSF-TPPA is negative, it is almost impossible for the patient to have neurosyphilis [9]. The original intention of this study was to reduce the rate of missed diagnosis in neurosyphilis patients, and combined with the current diagnostic environment, the qualitative analysis of CSF-TPP was taken as one of the diagnostic conditions.

Although lumbar puncture and CSF examination are common methods for the diagnosis of NS, they are invasive, require patients to overcome their fear prior to puncture, and keep them in a supine position for a lengthy period of time (6 h) after a puncture. As a result, many syphilis patients refuse to be reexamined on schedule after the initial puncture, resulting in a loss of follow-up. Additionally, it is impractical to do lumbar punctures on every syphilis patient since inpatient hospital capacity and the availability of medical resources are limited in comparison to the number of patients. It should be mentioned that, for the diagnosis of many central diseases or NNS patients with other central symptoms, lumbar puncture becomes the focus of identification, so the demand for lumbar puncture becomes more urgent. Therefore, this study developed a risk score model for HIV-negative syphilis patients. The timing of lumbar puncture was proposed, and the strategy of lumbar puncture was optimized to reduce the missed diagnosis rate of NS under the condition of limited medical resources. Especially for ANS patients, timely screening can effectively prevent serious harm in the late course of the disease.

Logistic regression analysis showed that male gender, older age, and increased serum TRUST titer were independent risk factors for HIV-negative NS patients. The phenomenon that men promote the incidence of NS was mentioned in the study by Yao Xiao et al. [11]. The proportion of men in NS is larger than that of women, and the reason for the large proportion may be that the sexual activity of men is higher than that of women, which provides more opportunities for the transmission of TPA. With increasing age, the risk of NS increases, which does not rule out the lengthening course of syphilis patients leading to an exacerbation of the disease.

The analysis in this study did not take the patients’ disease courses into account. Although the disease’s concealment was not taken into consideration, many patients were unsure of their syphilis status and even neglected to pay attention to the rash and other clinical symptoms or seek medical care. Antibiotic misuse can also affect the patient’s disease progression, making it difficult to tell the difference between a new infection and a reinfection.

The importance of serum TRUST quantification as an NS predictor has been emphasized time and time again in earlier research. Patients with a serum TRUST titer of 1:16 or greater had a higher chance of developing NS than those with a titer of less than 1:16; this risk rose by at least eight times [12].

Previous studies have demonstrated that a 45-year-old male with a serum TRUST titer of 1:16 is more likely to develop NS in syphilis patients, and this cut-off point for lumbar puncture is recommended [13]. In some studies, a serum TRUST titer of 1:32 was considered the cut-off point [14–17]. When the aforementioned criteria were definitely met, the risk scoring model developed in this investigation gave a total risk score of 8, which corresponded to a predicted probability of NS in HIV-negative syphilis patients of 58.4%, which was in the center of the high-risk category. Think about how many patients were missed between the intermediate and high-risk thresholds (23.3% and 33.6%) and 58.4%.

It is more advantageous for patients to limit the missed diagnosis rate of NS as much as feasible and treat it as soon as possible given the seriousness of the prognosis, the significant harm that NS causes to the patient’s body, and the low cost of penicillin. Therefore, we considered making a new proposal based on previous studies on the indication of lumbar puncture.

### For syphilis patients in the high-risk group (6–11 points)

Lumbar puncture and CSF examination are strongly recommended to strictly identify NS.

### For syphilis patients in the medium-risk group (4–5 points)

A lumbar puncture should be performed when possible. Patients who failed to undergo lumbar puncture should be reexamined strictly and regularly according to the serum TRUST quantification after syphilis treatment and the risk score should be performed periodically.

### For patients in the low-risk group (− 1 ~ 3 points)

Serum should be reexamined regularly after treatment of syphilis.

The advantage of the risk scoring model is that it is not limited to the boundary points of independent risk factors. Instead, the total score and risk probability were obtained by adding the weight scores satisfied by the independent risk factors (age, gender, and serum TRUST quantitative segment) of syphilis patients, and the risk group was judged. The risk was assessed by integrating the interaction effects of risk factors, which could accommodate more possible cases. Thus, the probability of missed diagnosis in NS patients due to failure to reach a certain index can be reduced, and grouped diagnosis and treatment suggestions can improve the efficiency of diagnosis and treatment.

Since this is the first scoring model to be developed, it still needs to be optimized and updated with more clinical data and clinical practice.

## Conclusion

The risk scoring model in this study can classify the risk of neurosyphilis in syphilis patients, optimize the lumbar puncture strategy to a certain extent, and provide ideas for the clinical diagnosis and treatment of HIV-negative neurosyphilis.

## Data Availability

This article is original, and all research data are reflected in this article. Raw data can be obtained from the corresponding authors according to reasonable requirements.

## Abbreviations

HIV: Human immunodeficiency virus
ROC: Receiver operating characteristic curves
NNS: Non-neurosyphilis
TRUST: Toluidine red unheated serum test
TPPA: Treponema pallidum particle agglutination test
WBC: White blood cell count
Pro: Protein quantification
AUC: Area under the curve
WHO: World Health Organization
NS: Neurosyphilis
CSF: Cerebrospinal fluid
VDRL: Venereal Disease Research Laboratory
